# Zymosan Particle-Induced Hemodynamic, Cytokine and Blood Cell Changes in Pigs: An Innate Immune Stimulation Model with Relevance to Cytokine Storm Syndrome and Severe COVID-19

**DOI:** 10.3390/ijms24021138

**Published:** 2023-01-06

**Authors:** Gábor Kökény, Tamás Bakos, Bálint András Barta, Georgina Viktória Nagy, Tamás Mészáros, Gergely T. Kozma, András Szabó, János Szebeni, Béla Merkely, Tamás Radovits

**Affiliations:** 1 Department of Translational Medicine, Semmelweis University, 1089 Budapest, Hungary; 2 International Nephrology Research and Training Center, Semmelweis University, 1089 Budapest, Hungary; 3 Nanomedicine Research and Education Center, Department of Translational Medicine, Semmelweis University, 1089 Budapest, Hungary; 4 Heart and Vascular Center, Semmelweis University, 1122 Budapest, Hungary; 5 SeroScience Ltd., 1089 Budapest, Hungary; 6 2nd Department of Pediatrics, Semmelweis University, 1085 Budapest, Hungary; 7 Department of Nanobiotechnology and Regenerative Medicine, Faculty of Health Sciences, Miskolc University, 2880 Miskolc, Hungary; 8 Translational Nanobioscience Research Center, Sungkyunkwan University, Suwon 16419, Republic of Korea

**Keywords:** cytokine storm, pulmonary hypertension, systemic inflammation, inflammatory cytokines, cytokine storm, chemokines, white blood cells, granulocytes, lymphocytes, pigs, animal models, IL-6, IL-1beta

## Abstract

Hemodynamic disturbance, a rise in neutrophil-to-lymphocyte ratio (NLR) and release of inflammatory cytokines into blood, is a bad prognostic indicator in severe COVID-19 and other diseases involving cytokine storm syndrome (CSS). The purpose of this study was to explore if zymosan, a known stimulator of the innate immune system, could reproduce these changes in pigs. Pigs were instrumented for hemodynamic analysis and, after i.v. administration of zymosan, serial blood samples were taken to measure blood cell changes, cytokine gene transcription in PBMC and blood levels of inflammatory cytokines, using qPCR and ELISA. Zymosan bolus (0.1 mg/kg) elicited transient hemodynamic disturbance within minutes without detectable cytokine or blood cell changes. In contrast, infusion of 1 mg/kg zymosan triggered maximal pulmonary hypertension with tachycardia, lasting for 30 min. This was followed by a transient granulopenia and then, up to 6 h, major granulocytosis, resulting in a 3–4-fold increase in NLR. These changes were paralleled by massive transcription and/or rise in IL-6, TNF-alpha, CCL-2, CXCL-10, and IL-1RA in blood. There was significant correlation between lymphopenia and IL-6 gene expression. We conclude that the presented model may enable mechanistic studies on late-stage COVID-19 and CSS, as well as streamlined drug testing against these conditions.

## 1. Introduction

The cytokine storm syndrome (CSS), a hyper-inflammation characterized by abnormally high levels of proinflammatory cytokines and other immune mediators in blood, is known to be a major contributor to the death of patients with severe COVID-19 [1,2,3,4,5,6,7,8,9,10,11,12,13,14,15,16,17]. Among others, high serum IL-6 and IL-1RA levels were found as independent risk factors for mortality from COVID-19 [18,19,20,21,22,23]. CSS is, however, not the only sign of bad prognosis in late-stage COVID-19. Another one is the association of neutrophilia with lymphopenia, manifested in a rise in neutrophil/lymphocyte ratio (NLR) [24,25], and yet another is hemodynamic instability, including pulmonary hypertension [26,27,28] and/or systemic hypotension [29,30]. These symptoms can also arise in COVID-19 independent CSS [24,25,26,27,28]; thus, they are manifestations of the immune derangement, rather than direct consequences of the infection with SARS-CoV-2 virus.

A key piece of background information that provided rationale for this study was the fact that the hemodynamic derangement and WBC differential changes are also characteristic features of the pigs’ response to i.v.-administered nanoparticles [31,32,33,34,35]. The experimental setup, referred to as porcine complement (C) activation-related pseudoallergy (CARPA) model, has been used for the screening of nanoparticulate drugs (nanomedicines) for potential immune reactivity, manifested in infusion reactions [31,32,33,34,35]. The model utilizes zymosan, a strong activator of the innate immune system, as a highly reproducible positive control. Notably, zymosan’s robust pulmonary hypertensive and systemic hypotensive effects are observed essentially in all pigs regardless of whether they are responsive or not to the tested nanomedicines [32,33,34]. In the model, i.v. administration of a tiny amount of reactogenic nanoparticles induces prominent hemodynamic changes within minutes, including pulmonary hypertension, systemic hypotension, rise in heart rate, fall of cardiac output and ECG alterations. These transient changes may be associated with granulopenia, followed by reactive granulocytosis with or without thrombocytopenia, and skin alterations, such as flushing or a rash [31,32,33,34,35]. The above changes are easily measurable and are highly reproducible in different pigs, making the model uniquely useful for preclinical testing of acute allergic reactivity of nanoparticulate drugs or, in other words, the risk of anaphylactoid reactions [31,32,33,34,35]. This effect of zymosan in pigs, taken together with its capability to induce proinflammatory cytokines in murine models [36,37] and cultured human peripheral blood mononuclear cells (PBMCs) [38], led to the hypothesis that zymosan could be used to induce the above triad of prognostic endpoints of CSS in pigs, so that the use of the model can be extended to studying the mechanism and drug sensitivity of severe COVID-19 and other diseases involving CSS. Accordingly, the goal of the present experiments was to test if the adverse hemodynamic and hematological effects of zymosan could be associated with inflammatory cytokine release in pigs; if yes, the goal was to optimize the model to allow streamlined drug testing against CSS in severe COVID-19 and other diseases.

We observed that zymosan infusion triggered an acute pulmonary hypertension, followed by a prolonged and markedly increased neutrophil-to-lymphocyte ratio (NLR) in parallel with massive rise in IL-6, TNF-alpha, CCL-2, CXCL-10, and IL-1RA expression in blood, resembling COVID-19 associated severe cytokine storm syndrome.

## 2. Results

### 2.1. Early Hemodynamic, Hematological, and Immune Mediator Changes Caused by Low-Dose Bolus Injection of Zymosan

**Figure 1:**
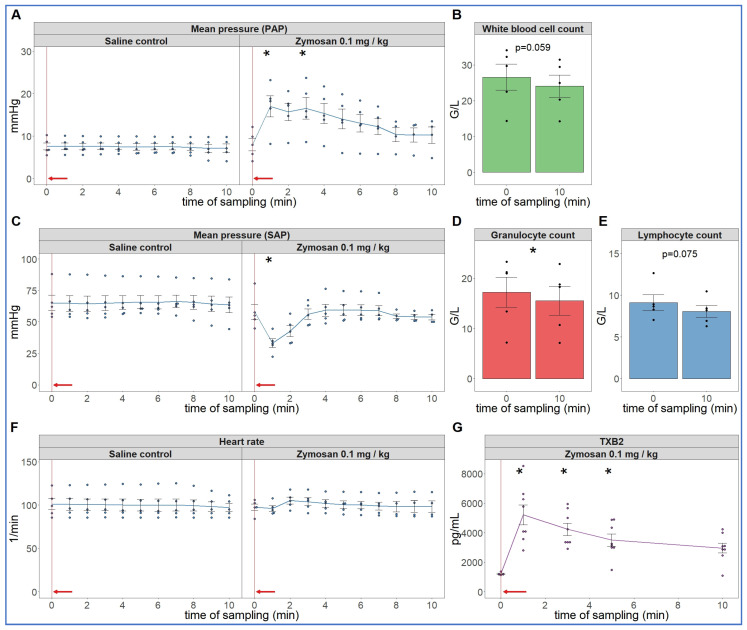
Physiological changes caused by bolus injection of 0.1 mg/kg zymosan in pigs. In (**A**,**C**,**F**) the PAP, SAP and HR were continuously recorded, and the coinciding values were averaged (±SEM) in four animals every minute over 10 min both before and after the zymosan injection. Zymosan administration is marked with red arrow. Blood white blood cell count (**B**), granulocytes (**D**) and lymphocytes (**E**) were counted in a coulter counter at 10 min after zymosan injection and were related to their respective baseline, i.e., the last blood collection before the zymosan injection (0 min). TXB2 increased 4-fold within a minute after zymosan administration (**G**). G/L means cell number × 10^6^/L, * *p* < 0.05 relative to respective baselines (0 min), determined using paired *t*-test.

As the first step in pursuing the hypothesis delineated in the introduction, we reproduced the robust hemodynamic changes caused by a single bolus injection of 0.1 mg/kg zymosan in pigs. As shown in Figure 1, the cardiopulmonary reaction starts with a sudden rise in pulmonary arterial pressure (PAP) (A), fall of systemic arterial pressure (SAP) (C) and massive release of thromboxane B2 (TXB2) (G) exactly paralleling the PAP. The heart rate (HR, E) and blood cells (B, D, F) showed no major changes, although a small, statistically significant decline of granulocyte count was detectable (D). The SAP returned to baseline within 10 min, while it took longer for PAP and HR (up to 30 min, not shown) to return to near normal levels.

### 2.2. Extended Follow-Up of Hemodynamic, Hematological, and Immune Mediator Changes Caused by High Dose Zymosan Infusion

**Figure 2:**
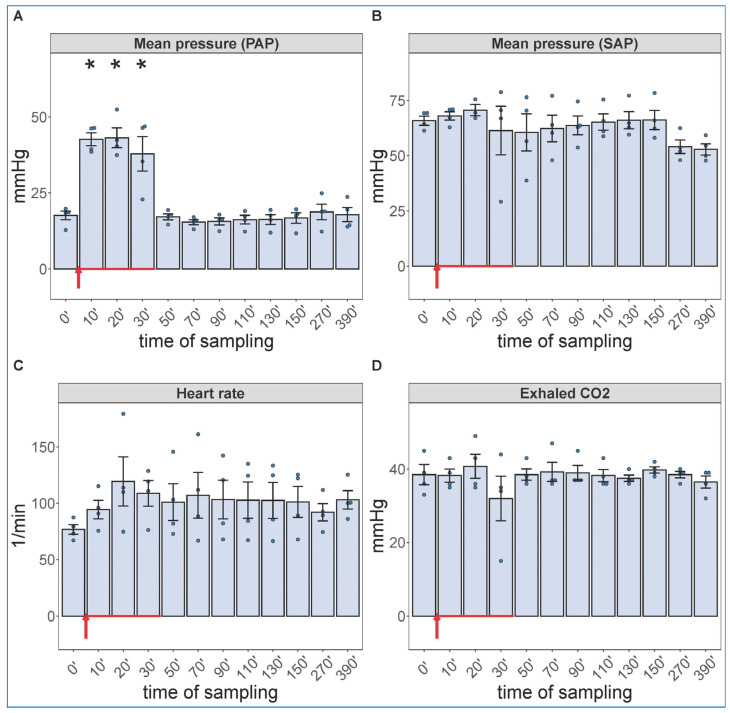
Physiological changes caused by infusion of 1.0 mg/kg zymosan in pigs. PAP (**A**), SAP (**B**), HR (**C**) and exhaled CO_2_ (**D**) were continuously recorded up to 6.5 h, and the coinciding values at the indicated time points were averaged (±SEM) in four animals. Here, we show the blood pressure on absolute (mmHg) scale without the flat pre-injection background (Figure 1). The red arrow indicates zymosan administration. * *p* < 0.05 relative to respective baselines (0 min), determined using paired *t*-test.

Due to a lack of major neutrophilia, the mentioned symptom of late-stage COVID-19 that we were trying to identify, we increased the administered dose of zymosan 10-fold and gave it in infusion, rather than bolus. Because the hemodynamic monitoring is invasive, this experiment had to be terminated after 6.5 h (390 min) observation period. Figure 2 shows the hemodynamic changes after initiation of the zymosan infusion. Interestingly, the infusion was associated with maximal rise in PAP; after completing it, however, the pulmonary pressure normalized within 20 min. The SAP showed major fall during infusion and then slow return to normal in 1 of 4 animals; this is a measure that has proven to be very variable in all previous CARPA studies [39,40,41,42,43,44]. The heart rate and exhaled CO_2_, an indicator of pulmonary function, showed no significant differences. These data imply an immediate cardiovascular effect of zymosan, which can be explained with immediate TXA_2_ release with entailing pulmonary vasoconstriction.

**Figure 3:**
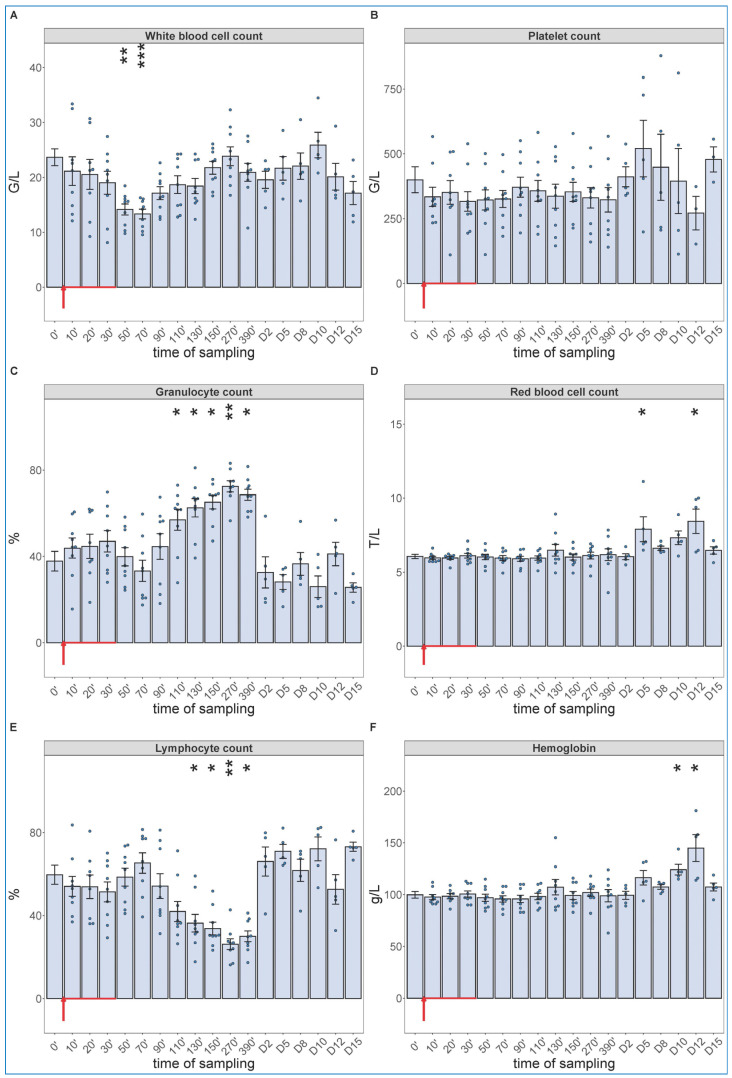
White blood cell count (**A**), platelet count (**B**), granulocyte % (**C**), RBC count (**D**), lymphocyte % (**E**), hemoglobin levels (**F**) at different sampling times. Zymosan administration is marked with red arrow. Until 390 min, the data have been merged from the 2nd (*n* = 4 pigs) and 3rd phase (*n* = 5 pigs) of the study, and their overlapping excludes a pro-inflammatory influence of hemodynamic monitoring (surgery). Kruskal–Wallis test with Dunn’s multiple comparisons post-hoc test, compared to respective baseline (0’) controls, * *p* < 0.05 vs. 0’; ** *p* < 0.01 vs. 0’; *** *p* < 0.001 vs. 0’.

Of particular importance regarding the hypothesis of this study, the blood cell changes did show the expected granulocytosis with lymphopenia, between about 1 and 6 h after starting the 30 min infusion. This effect is shown in Figure 3, together with data from five more pigs, which were not subjected to invasive blood pressure recording and, thus, were not sacrificed after 6.5 h. These animals were subjected to blood withdrawals for blood cell counting and cytokine analysis for up to 15 days, the results of which are presented below. The lack of significant changes in SAP, HR and exhaled CO_2_ suggests that these processes, in case of slow access of zymosan to blood, may be independent of pulmonary hypertension.

### 2.3. Long-Term Follow-Up of Hematological, TXB2 and Cytokine Changes Caused by High Dose Zymosan Infusion

As shown in Figure 3, we observed a significant drop in WBC count around 1 h after the start of zymosan infusion (Figure 3A), which did not involve changes in neutrophil granulocyte/lymphocyte ratio (NLR, about 4/6) (Figure 3C,E). However, thenceforwards, while the WBC count returned to normal in about 3–4 h (Figure 3A), the NLR gradually rose up to 7/3, near 3–4-fold relative to the baseline ratio. In absence of absolute (total) increase or decrease in WBC count at the peak of NLR (Figure 3A,C,E), the above changes imply a relative leukocytosis with absolute lymphopenia, a shift in WBC differential in favor of the innate, nonspecific versus the acquired, specific antimicrobial immune response. The platelet count did not change over time (Figure 3B), and we did not measure consistent trends in RBC counts or blood hemoglobin levels either, although the numbers showed significant rises relative to baseline on some days (Figure 3D,F).

**Figure 4:**
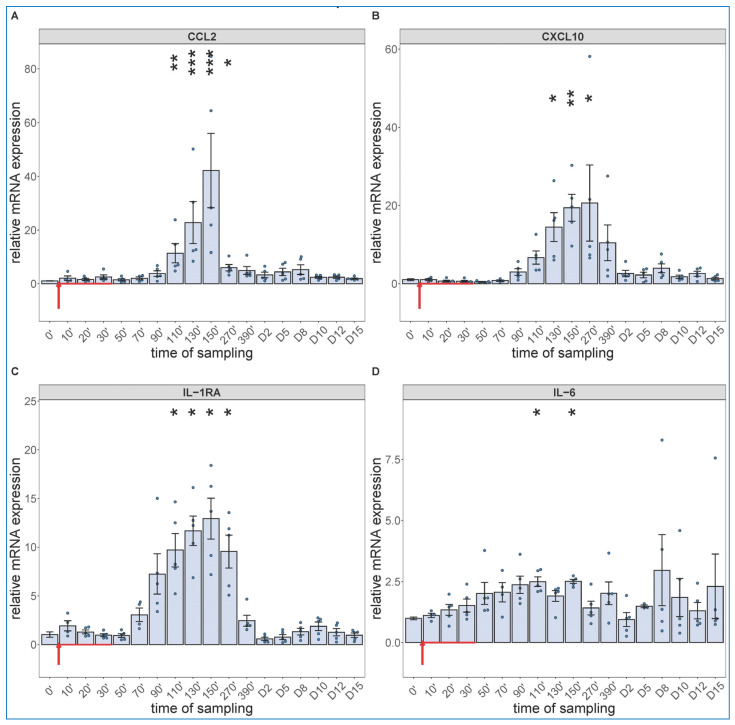
Gene expression of CCL2 (**A**), CXCL10 (**B**), IL-1RA (**C**) and IL-6 (**D**) were assessed from PBMCs isolated at different sampling times and normalized to 18S rRNA. Zymosan administration is marked with red arrow. Data are expressed as fold expression relative to a baseline control sample (0 min). Kruskal–Wallis test with Dunn’s multiple comparisons post-hoc test, compared to respective baseline (0’) controls, * *p* < 0.05 vs. 0’; ** *p* < 0.01 vs. 0’; *** *p* < 0.001 vs. 0’.

Regarding the cytokine changes, the PCR assay showed significant expression of CCL-2 (Figure 4A), CXCL10 (B), IL-1RA (C) and IL-6 mRNAs in close parallelism with the changes in WBC differential, with peaks in the 90–270 min range. The relative mRNA expressions decreased in the above order, while in terms of speed, IL-6 mRNA had faster rise than those of the other 3 cytokines (peaking at already 90–110 min vs. 130–150 min). We also detected significant increases in IL-6 gene transcription in 2 of 5 animals on day 8 and 10, again (Figure 4D).

**Figure 5:**
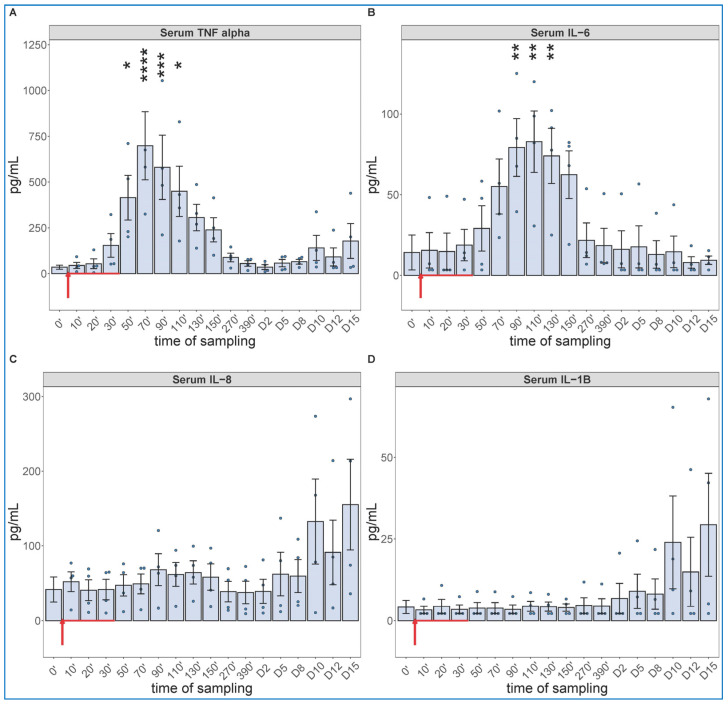
Serum TNF-alfa (**A**), IL-6 (**B**), IL-8 (**C**) and IL-1B (**D**) levels at different sampling times (ELISA). Zymosan administration is marked with red arrow. ANOVA, with Dunnett’s multiple comparison test, compared to respective baseline (0’) controls, * *p* < 0.05 vs. 0’; ** *p* < 0.01 vs. 0’; *** *p* < 0.001 vs. 0’; **** *p* < 0.0001 vs. 0’.

The cytokine protein assays performed for IL-6 and others (whose mRNA expression were not analyzed, i.e., TNF-alpha, IL-8 and IL-1beta) confirmed the early expression of IL-6 but showed the production of TNF-α to be even faster and more intense, peaking about 30 min earlier at about 10-fold higher concentration than IL-6 (Figure 5A,B). The concentrations of IL-8 and IL-1beta tended to be increased in some animals after day 10, with substantial individual variation (Figure 5C,D).

**Figure 6:**
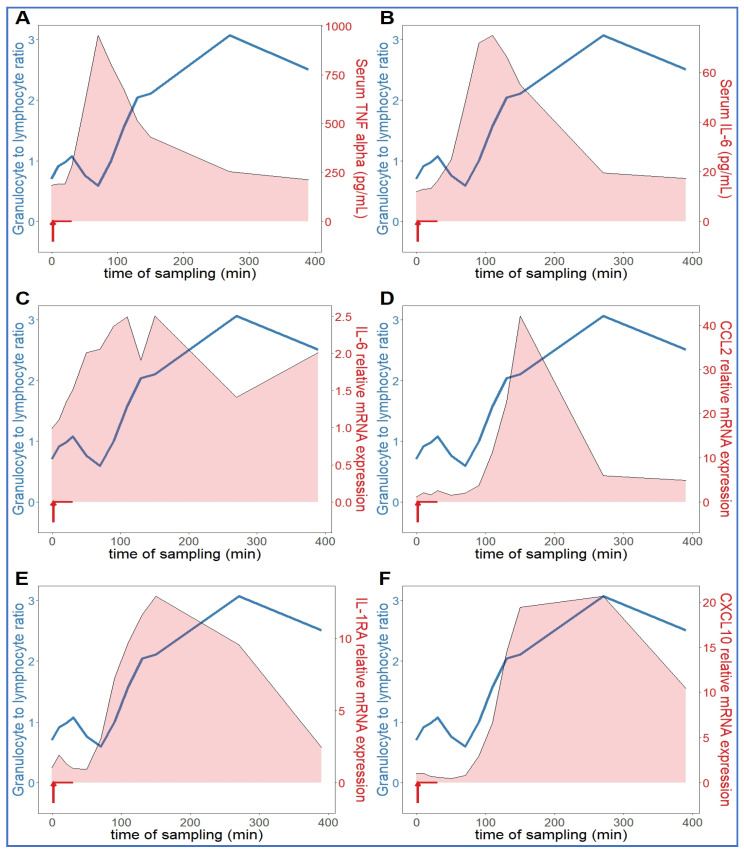
Relationship between serum cytokine levels and NLR. The NLR (neutrophil granulocyte-to-lymphocyte ratio (blue line) increased continuously in an hour after zymosan administration, which was preceded by increased TNF-alpha (**A**) and IL-6 (**B**,**C**). The expression of CCL2 (**D**), IL-1RA (**E**) and CXCL10 (IP-10, (**F**)) genes proceeded in parallel with the rise in NLR (*n* = 9).

**Figure 7:**
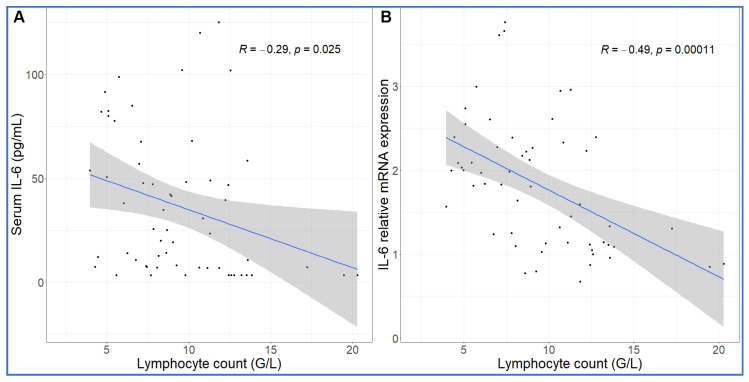
Correlation between IL-6 protein secretion and gene expression and absolute lymphocyte counts. The lymphopenia significantly correlated with increased IL-6 protein (**A**) and mRNA expression (**B**) over time. Linear regression.

Comparing the kinetics of NLR changes with cytokine transcription or translation revealed overlays with all cytokines analyzed (Figure 6A–F), except IL-8 and IL-1beta, which did not show changes in the 30–270 min period (Figure 5C,D). Figure 6A–F also shows that the rise in NLR, which reflects a shift in cellular immune response towards innate defense, started after about 1 h and peaked at 5h (blue line in Figure 6A–F). The cytokines whose early rises and peaks consistently preceded these changes of NLR were TNF-α and IL-6 (Figure 6A–C). In fact, for IL-6, we could show highly significant correlation between lymphopenia and IL-6 mRNA expression (Figure 7). Nevertheless, it would be premature to point to certain cytokines versus others as sole or most significant contributors to the blood cell changes, since there were also overlaps between the changes in NLR and expression of CCL2, IL-1RA and CXCL-10 genes (Figure 6D–F). Notably, we do not know the individual contribution of different cytokines to the blood cell changes. Thus, what can certainly be stated is that cytokine production correlated with the rise in NLR, which is consistent with the coincidence of NLR rise and CSS in severe COVID-19.

## 3. Discussion

### 3.1. The Structure and Immune Effects of Zymosan

**Figure 8:**
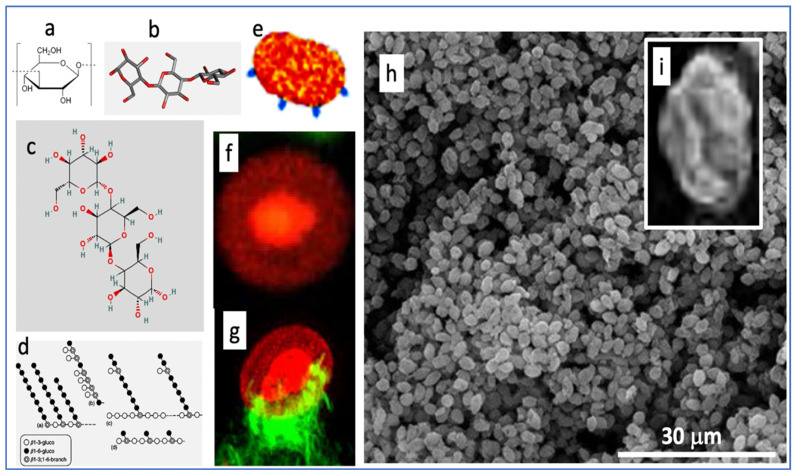
Various illustrations of zymosan. Panels (**a**–**d**) are chemical, graphical, and 3D-steric presentation of structure, (**e**) is an artistic visualization and (**f**–**i**) are electron microscopic and scanning electron microscopic images of zymosan. The corona in (**f**) is with a fluorescent dye conjugated to zymosan, and (**g**) is a zymosan particle undergoing phagocytosis, where the green fluorescing “claws” are macrophage pseudopods embracing the particle as first step of phagocytosis. The insert (**i**) is a particle zoomed in from (**h**). Free pictures from the internet and modified reproductions of figures in Ref. [47] with permission.

To help understand how a short infusion of yeast microparticles (zymosan) can reproduce three key manifestations of end-stage COVID-19 within 6 h, we summarize here the unique features of zymosan. It is extracted from the membrane of *Saccharomyces cerevisiae* and consists of mannosylated cell wall proteins and highly branched beta-glucans. The latter are D-glucose polymers linked by 1,3- and 1,6-beta-glycosidic-bonds. Zymosan is widely used in immunological studies as a powerful stimulator of innate humoral and cellular immunity. The chemical structures (Figure 8a–d) reveal little about zymosan’s real-life appearance (Figure 8e–i), i.e., 2–4 µm bean-shaped microparticles covered by knobs or bulges with extensions reminiscent of truncated bacterial pili [45,46].

Zymosan was described as a potent activator of the complement system 81 years ago [48] and has been used for modeling phagocytosis [46]. Similarly to Toll-like receptor (TLR)-4 mediated LPS stimulation of NF-kB in immune cells, zymosan stimulates the production of inflammatory cytokines via Toll-like receptors TLR-2 and TLR-6 [49,50]. Furthermore, zymosan activates another transmembrane signaling receptor, Dectin-1 [51,52,53], which collaborates with TLR-2 in NF-kB mediated cytokine production [54,55]. Due to all these redundant pro-inflammatory stimuli, zymosan has been used in many inflammatory disease models in mice and rats, as listed in Table 1.

### 3.2. Current Animal Models of CSS

Despite substantial efforts to develop effective pharmacotherapy against severe COVID-19, the standard of care today is based on traditional antibiotic and anti-inflammatory agents and some monoclonal antibodies, whose success is limited [73,74,75,76]. One contributing reason for the shortage of new, more specific, and effective drugs is the lack of an appropriate, widely accessible animal model of COVID-19 or CSS. Natural and genetically modified species used to model different aspects of COVID-19 include mice, ferrets, cats, dogs, pigs, and non-human primates [77,78,79,80,81]. The models described for CSS include the Staphylococcal superantigen mutant model in rabbits [82], the hemolytic transfusion model in mice [83], and the reactions of dogs to anti-CD28 mAb [84], primates to simian immunodeficiency virus [85], or pigs to a virulent African swine fever virus [86], yet another porcine model utilized LPS to induce CSS along with ARDS [87]. However, none of these models can recapitulate the sustained immunopathology of patients with severe COVID-19 or CSS. Moreover, the use of gene-modified animals and high-containment BSL3+ facility in the case of infectious virus are difficult to implement for high throughput drug testing in the pharmaceutical industry.

### 3.3. Molecular and Cellular Mechanisms of Zymosan’s Multiple Effects

As to why it is possible in pigs to reproduce three unfavorable disease markers in severe COVID-19 with zymosan, a likely answer is that zymosan is a very strong, multivalent stimulator of the innate immune system, a condition that also prevails in late stage COVID-19. Pigs, just as calves, sheep, goats, and some other species, are very sensitive to innate immune stimulation [88]. These species have pulmonary intravascular macrophages (PIM cells) in their lungs’ microcirculation, which firmly attach to the capillary endothelium via junction-like intercellular adhesion plaques [88]. The highly phagocytic and intense secretory PIM cells with direct access to blood are also close to the smooth muscle cell layer of blood vessels, making these animals’ pulmonary arteries highly sensitive to the vasoactive mediators liberated upon encounter with, and phagocytic uptake of nanoparticles. These include TXA_2_, a strong vasoconstrictor eicosanoid, which is the prime suspect in the hemodynamic changes caused by i.v. nanoparticles in pigs [88]. Zymosan can stimulate these macrophages via three independent pathways: one is via the anaphylatoxin (C3a, C5a) receptors, another is via TLR-2/6, and the third one is the Dectin-1 receptors [88]. These redundant activation pathways explain the high inter-animal reproducibility of hemodynamic changes caused by zymosan. Zymosan’s exertion of its vasoactivity and pro-inflammatory cytokine-inducing effects via cells exposed to plasma is supported by the finding that the kinetics of liberation of TXB2 and inflammatory cytokines in an in vitro peripheral blood mononuclear cell model of CSS [38] is very similar to those seen in pigs, namely immediate production of TXB2 and slower release of cytokines on a time scale of hours [38].

Regarding the lymphopenia and its correlation with IL-6 gene expression (Figure 7), IL-6 is known to upregulate the pro-apoptotic Fas, resulting in the loss of mature lymphocytes [89]. High levels of IL-6 might also reduce lymphocyte count through inhibition of lymphopoiesis in the bone marrow [90]. The neutrophil granulocytosis, in turn, is a common sign of strong inflammation with cytokine release, a well-known disease marker. As for the roles of CCL2 (C-C motif chemokine ligand 2, also known as monocyte chemoattractant protein 1, MCP1) and CXCL (C-X-C motif chemokine ligand 10, also known as interferon gamma-induced protein 10, IP-10), we have no information in the literature that would suggest a direct role of these chemokines in rising the NLR.

### 3.4. The Utility of Porcine Zymosan-Induced CSS Model

The experimental procedures applied in this study represent relatively straightforward in vivo investigation of systemic flare-up of inflammatory processes in the body, a complex immune phenomenon, a feared end-stage of many severe diseases including COVID-19, viral infections [91], monoclonal antibody and CAR-T-cell therapies [92,93], acute respiratory disease syndrome [94], and multiorgan failure [94,95]. CSS has multiple manifestations, and the different models discussed above focus on different endpoints. In the present model, we have focused on three standard physiological parameters which have been reported as bad prognostic indicators in late-stage COVID-19: pulmonary hypertension, rise in NLR and cytokine release, which can be also common features of all CSS, regardless of cause. This choice of endpoints, taken together with the increasing appreciation of pigs, as an immune toxicology model [34], the inexpensive access to zymosan, the rapid (up to 6 h) experimentation, the avoidance of problematic interpretation of immune data in murine models, exotic animals or infectious viruses with need for BSL-3 facility, or sophisticated gene technology, suggests that the porcine zymosan-induced CSS model may provide a new tool to better understand and develop effective pharmacotherapies against CSS in general, and end-stage COVID-19, in particular.

### 3.5. Outlook for the Pharmaceutical Industry

Quoting from a recent review by Cron [73], “more than 2 years into the pandemic, almost 6 million people have died from COVID-19 worldwide. Many people who succumbed to the virus had CSS”; however, there is “no perfect therapy” for this disease [73]. There is a clear need for R&D of new drugs, drug combinations, and perhaps new treatment approaches against CSS. The preclinical testing of a large number of drug candidates at different doses and different combinations could certainly be streamlined by using an in vivo single-treatment, relatively short-duration, reproducible large animal model requiring only a small number of animals and using a reasonably simple endpoint. The porcine model described in this study meets these conditions, and although the hemodynamic and cytokine changes were critical in realizing the relevance of the model for CSS and end-stage COVID-19, the third endpoint in this study, NLR, represents an automated routine laboratory blood assay that seems to be sufficiently quantitative and reproducible to provide a validatable disease marker without need for terminal surgery (for the PAP assay) or labor-intense qPCR and polyplex ELISA tests of cytokines. In addition, the animals may be reused after a washout period, which may help in ethical and financial aspects.

## 4. Materials and Methods

### 4.1. Materials

Ficoll-Paque was obtained from GE Healthcare Bio-Sciences AB (Uppsala, Sweden). The porcine C3a kit was obtained from TECOMedical AG (Cat No: TE1078, Sissach, Switzerland). Zymosan, Dulbecco’s phosphate-buffered saline (PBS) without Ca^2+^/Mg^2+^ were from Sigma Chemical Co. (St. Louis, MO, USA). Pre-designed primers for IP10 (CXCL10, Assay cID: qSscCED0019399) and IL6 (Assay ID: qSscCED0014488) were purchased from BioRad laboratories (Herkules, CA, USA).

### 4.2. Animals

Landrace pigs were obtained from the Research Institute for Animal Breeding, Nutrition and Meat Science of the Hungarian University of Agriculture and Life Sciences (Herceghalom, Hungary). The study involved 13 female and castrated male pigs in the 22–32 kg size range. The experiments were approved by the Ethical Committee of Hungary for Animal Experimentation (permission numbers: PE/EA/843-7/2020 and conformed to the EU Directive 2010/63/EU and the Guide for the Care and Use of Laboratory Animals used by the US National Institutes of Health (NIH Publication No.85-23, revised 1996).

### 4.3. Anesthesia and Instrumentation 

Animals were sedated with an intramuscular injection of 25 mg/kg ketamine (50 mg/mL, Gedeon Richter Plc. Budapest, Hungary) and 0.3 mg/kg midazolam (15 mg/3 mL, Kalceks AS, Riga, Latvia), and were carefully transported into the laboratory. Anesthesia was induced with a propofol bolus through an auricular vein. Airways were secured by inserting an endotracheal tube. Unless otherwise specified, animals were allowed to breathe spontaneously during the experiments. Controlled ventilation was applied in case of animals, where continuous measurement of hemodynamic parameters necessitated invasive surgical interventions. Surgery was done after povidone iodine (10%) disinfection of the skin. To measure the pulmonary arterial pressure (PAP), a Swan-Ganz catheter (Arrow AI-07124, 5 Fr. 110 cm, Teleflex, Morrisville, NC, USA) was introduced into the pulmonary artery via the right internal jugular vein. A Millar catheter (SPC-561, 6 Fr. Millar Instruments, Houston, TX, USA) was placed into the left femoral artery to record the systemic arterial pressure (SAP). Additional catheters were introduced into the left external jugular vein for drug administration, into the left femoral vein for venous blood sampling, and into the right common carotid artery for arterial blood gas analysis. The latter was executed with a Roche COBAS B221 benchtop analyzer (Roche Diagnostics, Rotkreuz ZG, Switzerland). Hemodynamic and ECG data were collected using instruments from Pulsion Medical Systems, and Powerlab, AD-Instruments (Castle Hill, Australia). Furthermore, end-tidal pCO_2_, ventilation rate and body temperature were also continuously measured, but they served no information beyond the measurements presented in this study and are therefore not shown.

### 4.4. Experimental Protocols in Different Stages 

This study was performed in three stages to measure the acute (minutes) and subacute (hours to days) effects of zymosan in animals dedicated only to this study and in others where zymosan was used as control. In the latter case, we used animals where other treatments caused no or minimal physiological changes, and it could be ascertained that the other treatments had no impact on zymosan’s effects. In the first stage, five pigs were treated with bolus injection of 0.1 mg/kg zymosan, and the resultant hemodynamic, hematological and blood immune mediator changes were monitored or measured as described below. In the second stage 1 mg/kg zymosan was infused in four pigs over 30 min, and the same protocol was applied as in stage 1, except that the monitoring and serial blood sampling lasted for 6.5 h. In the third stage, five pigs were infused with 1 mg/kg zymosan, followed by blood withdrawals at 10, 20, 30, min, then in increasing times until 6.5 h, and then at 2–3 days intervals for 15 days.

### 4.5. Blood Assays

As described above, 10 mL of venous blood was drawn from the pigs at different times into EDTA containing vacuum blood collection tubes (K3EDTA Vacuette, Greiner Bio-One Hungary, Mosonmagyaróvár, Hungary). 0.5 mL of blood was aliquoted for use in an ABACUS Junior Vet hematology analyzer (Diatron, Budapest, Hungary) to measure the following parameters of blood cells: white blood cell (WBC), granulocyte (GR) and lymphocyte (LY), platelet (PLT), red blood cell (RBC) count and hemoglobin (Hgb) concentration. For measuring thromboxane B2 (TXB2), a stable metabolite of thromboxane A2 (TXA2), 4 µg indomethacin (diluted in 2 μL of 96% ethanol) was mixed with 2 mL of anticoagulated blood to prevent TXA2 release from WBC before centrifugation at 2000× *g*, for 4 min at 4 °C. Another 2 mL of anticoagulated blood was directly centrifuged using the same settings to separate the plasma. After centrifugation, the plasma samples were aliquoted, frozen, and stored at −70 °C until the TXB2 assay was performed as described in the kits’ instructions. We used a commercially available ELISA kit (Cayman Chemicals, Ann Arbor, MI, USA) and an FLUOstar Omega microplate reader (BMG Labtech, Ortenberg, Germany).

### 4.6. Cytokine Measurements

Cytokine levels were measured in plasma samples derived from blood, which were taken in the previously discussed timepoints during and after zymosan infusion. The levels of IL-1beta, IL-6, IL-8 and TNF-alpha cytokines were determined using a high sensitivity 4-Plex Porcine Cytokine kit from Quansys Biosciences Inc. (Logan, UT, USA) according to the manufacturer’s protocol. Briefly, samples and standards were diluted with the provided sample diluent in 1:1 ratio. An eight-point calibration curve (7 points, 1 blank) was prepared using freshly suspended calibrator and standard serial dilutions were prepared ranging 2.13–1550 pg/mL for IL-1beta, 2.47–1800 pg/mL for IL-6, 2.88–2100 pg/mL for IL-8 and 2.19–1600 pg/mL for TNF-alpha. In a final volume of 50 microL, the calibration curve standards and samples were pipetted on a Q-Plex^TM^ Array 96-well plate. The plate was sealed, incubated for three hours, then washed with the provided wash solution, and the detection mix antibody solution was added. After 90 min incubation, the plate was washed; then, Streptavidin-HRP was added and incubated for another 20 min. After six final washes, the freshly prepared substrate solution was added and the plate was read immediately with 270 s exposure time using Imager LS by Quansys operated through Q-View Software (Quansys Biosciences, Logan, UT, USA), which was also used to evaluate the results. All samples and standards were measured in duplicate. All incubations were performed at room temperature (25 °C) on a shaker set to 500 rpm.

### 4.7. Isolation of PBMC and Quantitative RT-PCR

Peripheral blood mononuclear cells (PBMC) were isolated from 4 mL anticoagulated blood within 30 min at each experimental time point. Briefly, 2 mL blood was transferred into a 15 mL tube and diluted with 2 mL phosphate-buffer saline (PBS pH 7.4). In a new 15 mL tube, 3 mL Ficoll-Paque media (GE Healthcare, Chicago, IL, USA) was pipetted into the bottom and the diluted 4 mL blood sample was carefully layered on top, centrifuged for 30 min at 400× *g*. The upper plasma layer was removed, and the leukocyte layer was transferred into a new tube containing 6 mL PBS, washed and centrifuged. The PBMC pellet was resuspended in 1 mL TriZol (Thermo Fisher Scientific, Waltham, MA, USA) and total RNA was extracted according to manufacturer’s instructions. RNA pellet was resuspended in RNAse-free water and the RNA concentration was determined photometrically on a NanoDrop microphotometer (Thermo Fisher). One microgram of RNA of each sample was reverse-transcribed with the high-capacity cDNA reverse transcription kit from Applied Biosystems (Applied Biosystems/Life Technologies, Carlsbad, CA, USA) using random primers in a final volume of 20 μL. Quantitative real-time PCR reactions were performed on a Bio-Rad CFX96 thermal cycler (Bio-Rad Hungary, Budapest, Hungary) using the SensiFast SYBR Green PCR Master Mix (Thermo Fisher). The specificity and efficiency of each PCR reaction was confirmed with melting curve and standard curve analysis, respectively. Each sample was quantified in duplicate and normalized to the same sample’s 18S rRNA (RN18S) expression. Mean expression values were calculated as fold expression relative to a baseline control sample using the 2^−ΔΔCt^ formula. Pre-designed primers for IP10 (CXCL10, Assay ID: qSscCED0019399) and IL6 (Assay ID: qSscCED0014488) were purchased from BioRad. Primer sequences for RN18S, IL1RA and CCL2 are shown in Table 2.

### 4.8. Statistical Analyses

All data are presented as mean ± SD. Statistical analysis was performed using SPSS 10 (IBM, New York, NY, USA). Basic cardiopulmonary parameters were evaluated using paired *t*-test, while blood cell counts and PBMC gene expression values were evaluated using Kruskal–Wallis test and Dunn’s post-hoc test for multiple comparisons. Serum cytokine protein levels were evaluated with ANOVA followed by Dunnett’s multiple comparison test. Level of significance was set to *p* < 0.05 in each analysis.

## Figures and Tables

**Table 1:** Murine models of inflammatory diseases using zymosan as inflammation inducer.

Animal	Disease	Reference
mouse	irritable bowel syndrome	[56]
	T-cell-mediated autoimmune response	[57]
	allergy	[58]
	systemic inflammation	[59,60]
	arthritis	[61]
	sepsis	[62]
	air pouch model of inflammation	[63]
	multiple organ dysfunction syndrome (MODS)	[64]
	septic shock	[36]
rat	peritonitis	[65]
	arthritis	[66,67]
	air pouch model of inflammation	[68]
	chronic pelvic pain syndrome	[69]
	systemic inflammatory response syndrome (SIRS)	[70]
	peritonitis	[71]
	non-septic shock	[72]

**Table 2:** Primer sequences for qPCR analysis.

Gene Symbol	Forward Primer	Reverse Primer
ssRN18S (NR_046261)	GACAAATCGCTCCACCAACT	CCTGCGGCTTAATTTGACTC
ssIL1RA (NM_214262)	CAAGCCTTCAGAATCTGGGATGTC	GGCTCAACAGGCACCACATC
ssCCL2 (NM_214214)	GAAGCAGTGATCTTCAAGAC	GGGCAAGTTAGAAGGAAATG

## Data Availability

Experimental data are available upon reasonable request to the corresponding author.
